# A Treatise on the Etiology, Pathology, and Treatment of Congenital Dislocations of the Head of the Femur; Illustrated with Plates

**Published:** 1850-07

**Authors:** 


*A Treatise on the Etiology, Pathology, and Treatment of Congenital Dislocations of the Head of the Femur; illustrated with plates.* By John Murray Carnochan, M. D., Lecturer on Operative Surgery with Surgical and Pathological Anatomy, &c., &c. New York, S. S. & W. Wood, Publishers, 1850.

We are exceedingly obliged to the Publishers for this valuable monograph. The paper, the typography, the engraving and the *matter*, are all excellent: and it is withal very opportune, for until now no complete treatise on congenital dislocations of the hip joint was to be found in American or English literature. Neither Sir Astley Cooper, nor Sir Benjamin Brodie, both of whom have devoted especial attention to accidents and diseases of the joints, make any mention of this form of disease. In the English translation of Chelius we find a page or two given to the subject, but the laborious annotator, Mr. South, has not thought it worthy of a single paragraph. In France, however, it has recently attracted the attention of some of the most able surgeons and pathologists. Dupuytren wrote a treatise upon it as early as 1826, and since then, Breschet, Sedillot, Pravas, D'Outrepont, and Cruvelhier, the two latter of whom our author has omitted to mention, have continued the investigations with considerable zeal and success. The modern German writers, Von Ammon, Schreger, and Stromeyer, ought also not to have been forgotten as having contributed materially to the elucidation of its obscure pathology. It is to M. Jules Guerin, however, of Paris, that we are at present chiefly indebted, and from whose extensive researches, first published in the *Gazette Medicale*, in 1841, Mr. Carnochan has drawn the principal materials of his work.

Chapter *First* is devoted to general remarks upon the subject of which the author is about to treat. The varieties of congenital luxation of the head of the femur in regard to position are, says Mr. Carnochan, four, viz., upward, upward and forward, a subluxation upward and backward, and a complete luxation upward and backward, of which the last only admits of surgical relief, and is the subject of consideration in the present treatise, each of the other forms of displacement occurring only in monstrosities.

Chapter *Second* relates to the anatomical peculiarities of the hip joint, from the fifth to the ninth month, and is accompanied with an explanatory plate. The anatomy of the bones, muscles and cartilages, is described and delineated, and also, as necessary to a correct understanding of the etiology of the malady, the evolution of the excito-motor nerves is traced from their origin to their complete developement. In reference to the bones, it is observed that the three osseous portions of the acetabulum are gradually formed from as many separate points, so that at the middle period of intrauterine life, the period chosen for this description, three several spaces exist in the bony margin of the acetabulum. The space between the ilium and ischium is the most considerable, and opens towards the ischiatic notch, or the dorsum of the ilium, and it is through this gate that the head of the femur is supposed to escape; a circumstance which must be facilitated moreover, by the position which the legs of the fœtus usually occupy in utero, viz. flexed upon the abdomen, by the shallowness of the acetabulum, it being only about two lines in depth even including the ligamentous border with which the cavity is surmounted, and also by the action, either spasmodic or normal, our author believes it to be spasmodic, of the gluteii and other muscles.

The Etiology constitutes the theme of the *Third* chapter. Mr. Carnochan suggests in the outset that the term "spontaneous" as applied to this form of luxation, is indefinite, inasmuch as the same term is used to designate a species of luxation which occurs at a later period in consequence of scrofulous disease of the hip: he prefers, therefore, the term "congenital."

The luxation may be complete or incomplete. It may be single, existing only on one side, or double. We are reminded also of a "pseudo-luxation," in which no actual displacement exists, but in which, nevertheless, some of the signs are present. Our author now deliberately approaches the discussion of the various hypotheses by which surgeons have sought to explain the occurrence of these peculiar congenital luxations. This he regards as the great object of investigation, which being fully resolved, all the questions of pathology, prognosis, treatment, &c., will follow as easy and natural sequences. "*Felix qui potuit rerum cognoscere causas,*" he exclaims, and thence proceeds to discuss, seriatim, the five principal theories; all of which being at length, as he believes, satisfactorily disposed of, he presents a new, and as he trusts, a more rational hypothesis.

We must confess, however, that while we concede to our author in the disposal of this part of his subject much ingenuity, we have by no means been convinced that he has yet furnished us with a certain and invariable causation, nor that there is not much that is reasonable and probable in some of the older doctrines. Let us take care that we do not reject an opinion simply because it has upon it the mould of age, nor adopt a new opinion for no better reason than that it is the latest.

The notion entertained by Hyppocrates, that these dislocations are the results of falls, blows and other injuries or accidents to the mother, while the child is in utero, is certainly "not probable;" but it is by no means certain that the violent contractions of the uterus while in labour, while the thighs of the infant are forcibly flexed upon its abdomen, may not produce a backward luxation. It is possible, therefore, that in some instances such uterine contractions, or possibly the hands of the accoucheur, making traction upon the groin of the infant, may constitute the sole cause.

The hypothesis of Dupuytren meets with still less favor from Dr. Carnochan. "Original defect in the organization of the germ," or "aberrations of the formative power," is an explanation "vague and inexplicit," says Dr. C., and it "has no support from physiological facts or the acknowledged doctrines regarding embryogeny." If we are not mistaken in what Dupuytren intended to teach, his hypothesis does not differ except in words from that put forward by M. Breschet, but to which Dr. C. has assigned a separate section. "Arrest of developement of the cotyloid cavity" is the more intelligible and significant explanation of M. Breschet, which doctrine is sustained by many parrallel facts in the history of developement.. We see daily numerous defective congenital formations, such as supernumerary fingers or toes, hare-lip, fissure of the palate, &c., which will admit of no better explanation than "aberration of the formative power," or "arrest of developement."

Neither can we find in the pathological announcement of M. Cruveilhier, such evidence against M. Breschet as should have led our author to exclaim exultingly, "Now, it so happens, that the cotyloid cavity and the head of the femur have both been found progressing to their normal developement, or to have completed it, in cases of this dislocation which have come under observation." It is not claimed that such a condition of matters has been found in *all* cases, and it is therefore fair to infer that in these exceptional instances, (we are not certain that they are only *exceptional*,) the cause might have been found in "arrest of developement." But Breschet is entitled to a more complete argument from this pathological fact discovered by M. Cruveilhier, and now adduced by Carnochan. It is positively asserted that the bones of the hip joint have in these cases been found "*progressing* to their normal developement." A careful reading will show that it is intended to say that the bones have not yet attained that growth and developement which would correspond with the age of the child, in short, he speaks here of an "arrest of developement."

"Certain articular maladies occurring in the fœtus during intrauterine life," have produced, according to M. Parise, the malady in question. We shall dismiss this supposition with the simple statement of our belief that according to the evidence presented in the symptoms and autopsies, it must be one of the least frequent causes, if indeed it can ever properly be assigned.

Finally, M. Guerin teaches that it is attributable to a "primitive alteration in the nervous centers," while the author concludes that it depends upon a "perverted condition of the medulla spinalis," and upon this the main issue is taken.

The question between M. Guerin and Mr. Carnochan, as we understand it, is this. Guerin attributes the deformity in question to a *pathological* process in the *nervous centers*—the medulla spinalis or encephalon—in consequence of which portions of these structures are removed, and hence certain muscles dependent upon this *center* for their energy, fail to develop: while Carnochan regards the medulla spinalis either as suffering irritation simply, or "perverted action," or as the center of the excito-motor system *through* which *external* agencies reflect their injuries upon these muscles, and cause them to contract and expel the bone from its socket. The one also regards the muscular shortening as a mere arrest of growth; the other, as a contraction consequent upon irritation, or perverted action.

Now as to the first, it is much more reasonable to suppose with Carnochan, that the absence of portions of the encephalon, or medulla spinalis, whether in connection with deformities or not, is due to arrest of developement, rather than to a pathological process. Nor does Guerin present one particle of evidence that in these cases any such process ever existed. It certainly cannot be found in the following passage.

"But if you consider that the specimens and the plates, now under view, show all the degrees of destruction of the nervous centres following in a decreasing series, which commences at the complete disappearance of the encephalon and the spinal marrow, and closes with a simple lesion of the membranes, you will easily understand by what chain of facts and of inductions I have been enabled to establish rigorously, the reality of this destruction in those cases, where we no longer find the union of all the characters which appertain to it." p. 67; yet this passage contains the only proof of the position which is offered, as to the rest it is mere assertion: and we could easily present cases from our own experience in disproof of the assertions.

Whether the second position of Guerin is correct, viz., that the muscles in question have not contracted, but were actually never developed, is altogether another issue, in which it seems to us that he has the right, or in which, to say the least, Guerin is as likely to be correct as Carnochan. M. Guerin, however, had made it a corollary from his first position, and Mr. Carnochan having rejected this, seems to think it necesary to reject also the inference. But listen to the conclusions of our author, p. 86. "What this particular source of irritation or pathologic action—the necessary antecedent of morbid muscular retraction—may be, it is not easy to determine strictly. Whether it amounts to inflammation; or to *ramollissement;* or to vascular congestion of the cineritious matter, or of the white structure in contiguity with it; or simply to what is called nervous irritation, caused by pressure or otherwise; or whether it depends upon an abnormal accumulation of nervous fluid, which by some has been supposed to be as nocuous in regard to the nervous substance, as the accumulation of blood in an inflamed tissue is to the several structures of the body; or whether from some poisonous condition of the blood—the nervous tissue bathed by this fluid as it circulates becoming morbidly impressed;—or whether an imperfect development of a portion of the medulla spinalis, or an atrophy of it, be essential in producing this disturbance of the excito-motor apparatus, are points which can only with great difficulty be decided in a rigorous manner." If, then, this "perverted action" may be described under all of these various morbid conditions, including "imperfect developement of a portion of the medulla spinalis, what reasons can be given why the muscles dependant upon this center may not also be imperfectly developed, and thus no occasion may exist to invoke active contraction or spasm as the cause?

In conclusion we cannot say that in the elucidation of the etiology of this disease, Mr. Carnochan has added much to what was hitherto known, or that he has done much towards clearing up and reconciling the obscurities of other theorists.

Here we shall leave the book for the present; the succeeding chapters, treating respectively of the Symptomatology, Diagnosis, Prognosis, Pathology and Treatment, are more free from speculation, and will well repay the student for the reading.

